# Intervention Using Low-Na/K Seasonings and Dairy at Japanese Company Cafeterias as a Practical Approach to Decrease Dietary Na/K and Prevent Hypertension

**DOI:** 10.3390/nu17243856

**Published:** 2025-12-10

**Authors:** Nagako Okuda, Aya Higashiyama, Kozo Tanno, Yuki Yonekura, Makoto Miura, Hiroshi Kuno, Toru Nakajima, Eiji Tahara, Fukiko Morimoto, Kozue Sugio, Kyoko Kojima, Tomomi Nagahata, Hirokazu Taniguchi, Akira Okayama

**Affiliations:** 1 Division of Applied Life Sciences, Graduate School of Life and Environmental Sciences, Kyoto Prefectural University, Kyoto 606-8522, Japan; nagahata@kpu.ac.jp; 2 Department of Hygiene, Wakayama Medical University, Wakayama 641-8509, Japan; ayahiga@wakayama-med.ac.jp; 3 Department of Hygiene and Preventive Medicine, Iwate Medical University, Yahaba 028-3694, Japan; ktanno@iwate-med.ac.jp; 4 Department of Nursing Informatics, Graduate School of Nursing Science, St. Luke’s International University, Tokyo 104-0044, Japan; yyonekura@slcn.ac.jp; 5 Collaborative Research Programs of SynCrest Inc., Iwate University, Morioka 020-8550, Japan; mako@iwate-u.ac.jp; 6 Nichinan Kogyo, Co., Ltd., Nikaho 018-0411, Japan; hkuno@kikkonan.co.jp; 7 Research and Development Division, Shoda Shoyu, Co., Ltd., Tatebayashi 374-8510, Japan; tnakaji@shoda.co.jp; 8 Nippon Paint Corporate Solutions Co., Ltd., Osaka 531-0077, Japan; eiji.tahara@nipponpaint.jp; 9 Nippon Paint Health Insurance Union, Osaka 531-8511, Japan; fukiko.morimoto@nipponpaint.jp (F.M.); kozue.sugio@nipponpaint.jp (K.S.); 10 The Research Institute of Strategy for Prevention, Tokyo 103-0006, Japanaokayama@jrisp.com (A.O.); 11 Faculty of Agriculture, Ryukoku University, Otsu 520-2194, Japan; hirokazu-t@agr.ryukoku.ac.jp

**Keywords:** lower-sodium salt substitute, potassium, blood pressure, company cafeteria, urinary Na/K

## Abstract

**Background/Objectives**: Reducing sodium (Na) and increasing potassium (K) intake are important for lowering blood pressure (BP). Practical measures to decrease the dietary Na/K ratio are needed. A crossover design intervention study serving lunches prepared using K-enriched (low-Na/K) seasonings and dairy at an employee cafeteria was conducted. **Methods**: Participants consumed lunches containing low-Na/K seasonings and dairy as intervention meals and regular lunches as control meals for four weeks each, and we examined the changes in BP and urinary Na/K ratios. The Na and K contents of the meals were also measured. **Results**: A total of 166 employees (17.5% of whom were hypertensive, while 42.2% were women; mean age of 44.3 years) who regularly use the cafeteria completed the whole survey. The intervention meals contained less Na (66.4 vs. 74.2 mmol/serving) and more K (15.1 vs. 9.9 mmol/serving) than the control meals, and the average Na/K ratio was significantly lower (4.6 vs. 8.2, *p* < 0.001). The average urinary Na/K ratio was significantly lower in the intervention period than in the control (3.69 vs. 4.10, *p* = 0.008), but there was no difference in the average BP. Participants rated both the intervention and the control meals to be similarly good; 69.5% and 73.7% reported that the taste was fine, respectively, with no significant difference. **Conclusions**: Employing low-Na/K seasonings and habitual dairy consumption at lunchtime were effective in reducing urinary Na/K in a healthy working population. This may be a practical measure for sustainably decreasing dietary Na/K and controlling age-related increases in BP. UMIN-CTR registration: UMIN000050876

## 1. Introduction

Hypertension is the major risk factor for cardiovascular disease (CVD) in many countries, and its prevention is critical [1,2]. In Japan, more than 70% of men and women aged over 70 years are hypertensive [3], and hypertension accounts for the largest contribution to CVD [4,5]. Despite the high prevalence in the elderly, the prevalence was 11% for men and 6% for women in their 30s in 2019 [6]. Because of its close association with aging, an effective population-scale approach that reaches young normotensives should be explored. In 2012, the World Health Organization (WHO) announced a target value of 5 g/day for salt (NaCl) consumption [7] and 3500 mg/day for K consumption [8] in adults. However, NaCl intake is much higher, and K intake is far less than the target values, resulting in high overall Na/K ratios.

Although the blood pressure (BP)-lowering effect of a low-Na/K diet has been demonstrated in previous studies [9,10,11,12], practical methods that can be applied to populations have not been well established. Salt reduction is generally considered to be a cost-effective measure [13], but it is difficult for many people to maintain long-term practice due to their salt preferences [14]. In health education, it is often recommended to eat more fruits and vegetables as sources of K intake. However, a meta-analysis of behavioral interventions showed that the increase in fruit and vegetable intake only reached an average of 0.31 portions/day [15]. Moreover, people with low socio-economic status generally eat less fruit and vegetables [16,17]. Hence, simply advocating for the consumption of these foods may have a limited effect. A practical solution must be implemented without requiring individuals to make additional effort to accomplish sustained lower dietary Na/K levels.

K-enriched seasonings (low-Na/K seasonings) in which a portion of the NaCl is substituted with K salts can be used to decrease dietary Na/K levels [18], and previous intervention studies using low-Na/K salt conducted in Taiwan and PRC have reported favorable effects on BP and CVD outcomes [19,20,21]. Regarding the possibility of complications due to an increase in K intake, there was no significant increase in adverse events, including clinically problematic hyperkalemia [20]. Thus, the use of low-Na/K salt may be a promising approach clinically and financially. We should also consider increasing K intake by adopting foods that are naturally rich in K. Milk and yogurt can be a good source of K (300 mg per cup), which can be consumed without the need for Na-containing seasoning or cooking, and the price is also relatively stable. In terms of convenience, dairy may be more effective than vegetables and fruits as an intervention food. Notably, the average intake of dairy has been lower in Japan than in Western countries; the average total dairy consumption for adults was reported to be 1.5 cups equivalent/day in the US [22] and 348 g/day in the Netherlands [23], whereas the average intake of dairy products among middle-aged adults in Japan was shown to be 126 g/day, providing 199 mg/day of K, which accounted for 7.8% of their total K intake [24].

The previous intervention studies enrolled primarily older, high-risk individuals [19,20,21]. If the dietary Na/K ratio of the younger healthy generation can be effectively and sustainably lowered, the preventive effects against hypertension throughout the entire life course may be more significant, considering that a lower dietary Na/K ratio at a young age may suppress subsequent increases in BP [25,26]. The feasibility and impact of using low-Na/K seasonings in the younger generation should be examined. Moreover, the acceptance of meals prepared with low-Na/K seasonings in populations with different dietary habits may be important. In previous intervention studies conducted in populations consuming Chinese cuisine, only low-Na/K salt was used as the intervention seasoning [19,20,21]; however, other seasonings besides salt may be necessary for some populations with different dietary habits to achieve effective and sustainable improvements in dietary Na/K ratios. The primary sources of Na intake differ by population. The INTERMAP Study reported that in China, 75.8% of Na intake came from salt added during cooking, while in Japan, 20.0% came from soy sauce, 9.7% from miso, 9.5% from salt, and 6.7% from noodle soup [27]. The effectiveness of low-Na/K seasonings should be examined using seasonings appropriate for the dietary habits of the target population. It should be evaluated whether such a change in seasonings can be implemented without a decrease in palatability or increased labor of kitchen chefs, thus ensuring sustainability.

In this study, we utilized low-Na/K seasonings, including soy sauce, miso, and noodle soups, which are primary Na sources in Japanese cuisine, and dairy as intervention foods in Japanese company cafeterias in a crossover intervention study and compared the BP and urinary Na/K levels between the intervention and control periods. We also examined whether the taste of the intervention and control meals differed, according to the participants’ evaluations.

## 2. Materials and Methods

### 2.1. Setting

**Figure 1:**
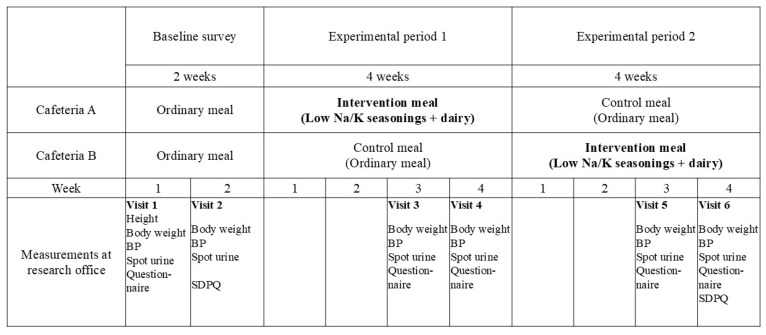
Outline of the intervention study. The research office was open on Mondays, Tuesdays, Thursdays, and Fridays. BP, blood pressure; SDPQ, short dietary propensity questionnaire.

The intervention study was conducted in two company cafeterias located on the same premises in Osaka, Japan. The total number of employees was 742 in December 2022. Of the two cafeterias, Cafeteria A was primarily used by factory employees and Cafeteria B by head office employees. Each cafeteria user usually used one of the cafeterias and did not alternate between the two. A food service company operated the two cafeterias, and the two cafeterias served the same lunch menus every weekday (five days per week). Figure 1 provides an overview of the study. The intervention diet was served at Cafeteria A during the first half of the experimental period and at Cafeteria B during the second half. The intervention periods were set to 4 weeks because the DASH-sodium study reported that the effects of dietary intervention on BP appeared after 2 weeks [12]. This study was approved by the ethics review boards at the Research Institute of Strategy for Prevention (No. 3-1), Kyoto Prefectural University (No. 240), and Wakayama Medical University (No. 3554).

### 2.2. Participants

**Figure 2:**
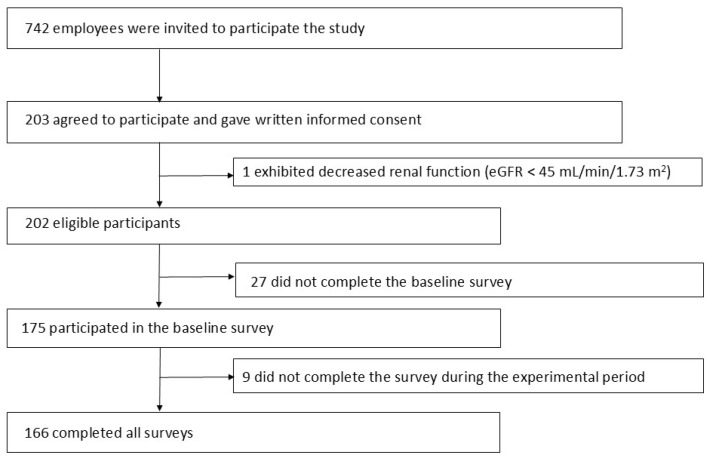
Recruitment of the study participants and their participation in the surveys.

Participants were recruited from the pool of employees who habitually used one of the two cafeterias in December 2022, when 50% of the employees were asked to refrain from coming to work due to the COVID-19 pandemic, and the number of people who regularly used the cafeteria had also decreased. After several postponements from the originally planned schedule in 2020, the intervention was eventually implemented between January and March 2023. Those who were instructed by their physicians to restrict K intake or those with decreased renal function according to regular health checkups (serum Cr ≥ 1.5 mg/dL and/or eGFR < 45 mL/min/1.73 m^2^) were excluded (Figure 2). Written informed consent was obtained from all participants.

Regular cafeteria menus were used, not menus specially created for the study. During the study period, the same menus were served at Cafeterias A and B, but with different seasonings. For the intervention meals, low-Na/K seasonings (reducing 25% of the NaCl and adding K salt) were used, and dairy was provided. Details of the seasoning selection were previously published [28]. Soy sauce, miso, ponzu (soy sauce with citrus juice), salt, and five types of noodle soups were replaced with low-Na/K types for the intervention meals. Two menus using low-Na/K seasonings were provided in the intervention period: a Japanese-style set menu (main dish, miso soup, and rice) and noodles. When changing seasonings, maintaining flavor may be one factor contributing to long-term sustainability and its subsequent health-promoting benefits. We asked the cafeteria manager to determine the amount of low-Na/K seasonings required to make the meals taste the way they normally did. We did not ask him to measure the amount of the seasonings for the purpose of using the same amount as the ordinary seasonings.

A refrigerator stocked with dairy products (regular milk, low-fat milk, and low-fat yogurt) was placed in the cafeteria. Participants were asked to select one of the two low-Na/K menu items and a dairy product for lunch on weekdays (Monday to Friday). There was no excess cost to the participants for the intervention meal and dairy. For eating outside of the cafeteria, including breakfast and dinner, we told the participants to eat as usual, with no restrictions. Because participants engaged in various job roles, they sometimes could not use the company cafeteria due to outings or business trips. We informed them that no restrictions would be placed on meals taken outside the cafeteria on such occasions. Menus using ordinary seasonings were also offered during the intervention period for cafeteria users other than the participants who preferred ordinary meals.

### 2.3. Measurements of the Na and K in the Meals

During the experimental period of 8 weeks (40 business days), we sampled dishes from the two cafeterias, namely those cooked with ordinary seasonings (control meals) and low-Na/K seasonings (intervention meals). The dishes included the main dish for the Japanese-style set menu, the miso soup, and the soup and topping for the noodles. For measurements, after weighing each serving of each dish, approximately 3 times the amount of water was added, and the total mixture was thoroughly ground in a blender. The aqueous component obtained after centrifugation at 1500 g for 5 min was used for the measurement. Ion electrode instruments (LAQUAtwin Na-11 and LAQUAtwin K-11, HORIBA, Ltd., Kyoto, Japan) were used, performing a calibration using standard solutions for each sample. Each sample was preliminarily diluted within the range of a suitable concentration for the measurement (300–500 ppm), and the concentration was determined using the recovery method. Each sample was measured twice, and the average value was used as the final result. Thus, the Na and K contents per serving and Na/K ratio were calculated.

### 2.4. Outcomes and Measurements

The primary outcome was the systolic BP (SBP), which was taken 6 times from each participant (2 for the baseline survey, 2 for the first half of the experimental period, and 2 for the second half of the experimental period, Figure 1). BP was measured 3 times at every visit using an automatic sphygmomanometer (OMRON HEM-8712) after 5 min of rest. Trained observers performed all procedures, including the BP measurements and reviewing the survey forms. The observers were blinded as to whether participants were in the intervention period or the control period. The average of the second and third readings was used in the analysis. The secondary outcome was the urinary Na/K ratio, which was also obtained from each participant during 6 visits. Participants were asked to collect spot urine from the first morning urination and bring it to each visit. BP measurements and urine sample collection were conducted on four weekdays (Monday, Tuesday, Thursday, and Friday), with participants visiting the research office on one of these days. Since no dietary intervention was conducted over the weekend (Saturday and Sunday), the urine provided on Monday was from a non-intervention day, while the urine samples submitted on the other three days were basically from days with dietary intervention.

Other prespecified outcomes included Na and K content of the meals, frequency of use for the cafeteria meals and dairy, and palatability of the meals. To monitor the participants’ food choices, food choice sheets were given to the participants every day during the intervention period, and they indicated the menu and dairy products they had. The last questionnaire performed at visit 6 included a taste evaluation of the control meals and the intervention meals using a three-point scale: fine, strong, light. As confounders, the height, weight, treatment of hypertension, and dietary propensities based on a short dietary propensity questionnaire (SDPQ) [29] were investigated.

### 2.5. Sample Size and Statistical Analysis

Sample size calculations were performed using BP data from multiple BP measurements in previous studies [30,31]. We estimated that the urinary Na/K ratio would decrease by 1.0 following the intervention, with 5 intervention meals per week [28], and the mean difference in SBP between the intervention and control periods was estimated to be 1.0 mmHg with a standard deviation of 5.0 mmHg. The required total number of participants in the crossover intervention was calculated to be 400, to provide 80% statistical power with a two-sided significance level (α) of 0.05.

The mean of two measurements for urinary Na (mEq/L), K (mEq/L), and Cr (mg/dL) in each baseline, control, and intervention period was used to calculate the estimated 24 h urinary Na and K excretion [32], as well as the urinary Na/K ratio for the corresponding period. Continuous variables were tested using a t-test and categorical variables using a chi-square test or Fisher’s exact test. BP and urinalysis in the intervention and control periods were compared using paired t-tests. The McNemar–Bower test was used to compare proportions between the intervention and control periods. The significance level was set at less than 0.05 (two-sided). All statistical analyses were performed using SPSS v.25 (IBM Japan Corp., Tokyo, Japan).

## 3. Results

### 3.1. Characteristics of Participants

A total of 203 employees agreed to participate (Figure 2). Of those employees, 175 participated in the baseline survey, and 166 completed all surveys and measurements. The characteristics of the 166 participants (102 men and 64 women) who completed all surveys are shown in Table 1. The overall mean age was 44.3 years, with no difference between men and women. Among them, 28.4% of the men and 14.1% of the women had ever been diagnosed with hypertension, and 12.7% of the men and 4.7% of the women were treated with antihypertensives. Approximately two-thirds of them did not have the habit of consuming milk/yogurt every day.

### 3.2. Baseline Survey

The mean SBP was significantly higher in men than in women (120.6 ± 12.8 mmHg vs. 108.0 ± 16.2 mmHg, *p* < 0.001) (Table 2). Upon classification, including the users of antihypertensives, more than half of the BP measurements were normotensive (SBP < 120 mmHg and diastolic blood pressure (DBP) < 80 mmHg), and 10.2% of the measurements were hypertensive (SBP ≥ 140 mmHg and/or DBP ≥ 90 mmHg). The estimated 24 h urinary Na and K excretions were both significantly higher in men than in women, and the mean urinary Na/K (mmol/mmol) was similar for men and women (4.01 ± 1.89 vs. 4.20 ± 2.00, *p* = 0.549).

### 3.3. Na Content, K Content, and Na/K Ratio of the Cafeteria Meals Served

The results of the Na and K measurements for the meals served in the cafeteria are shown in Table 3. Mean ± SD for Na content (mmol/serving) was significantly lower in the intervention meals than in the control meals for the main dish from the Japanese set menu (40.0 ± 18.7 vs. 47.9 ± 23.6, *p* = 0.013) and the noodles (71.8 ± 14.7 vs. 80.4 ± 14.8, *p* = 0.008). The mean K content (mmol/serving) was significantly higher in the intervention meals than in the control meals for the main dish from the Japanese set menu (11.5 ± 3.5 vs. 8.4 ± 3.4, *p* < 0.001) and the noodles (11.8 ± 3.4 vs. 4.1 ± 1.5, *p* < 0.001). For miso soup, although the mean Na content was similar, the mean K content was significantly higher (3.5 ± 0.6 vs. 1.4 ± 0.2, *p* < 0.001). The Na/K ratios were significantly lower in the intervention meals than in the control meals for all types of dishes. Each dairy product contained around 4 and 8 mmol of Na and K per pack, according to the information provided by the manufacturer, and thus the Na/K ratio was around 0.5.

### 3.4. Cafeteria and Dairy Use

Participants used the cafeteria an average of 4.2 ± 1.0 times/week during both the control and intervention periods (Table 4). The frequency was higher in men than in women. According to the food choice sheets, the participants ate ordinary meals for approximately 10% of the cafeteria use, rather than the low-Na/K meals in the intervention period (Supplementary Table S1). During the intervention period, they reported having dairy every time they used the cafeteria (approximately 4 times/week, Table 4). However, 42.2% of the participants reported the frequency of milk/yogurt to be 2–3 cups/week or less in the SDPQ at the last visit (Supplementary Table S2). They chose low-fat yogurt for 85% of their cafeteria use and regular milk for 11%, which did not significantly differ by sex (Supplementary Table S1).

### 3.5. Measurements in the Control and Intervention Periods

Results from measurements in the control and intervention meal periods are shown in Table 4. There were no significant differences in the changes in SBP or DBP between the intervention and control periods. The mean urinary Na/K ratio ±SD (mmol/mmol) was significantly lower in the intervention period than in the control period (3.69 ± 1.76 vs. 4.10 ± 1.89, *p* = 0.008). The difference was significant in women (3.31 ± 1.39 vs. 4.02 ± 2.24, *p* = 0.005), but not in men (3.94 ± 2.13 vs. 4.15 ± 2.20, *p* = 0.226).

The mean estimated 24 h urinary Na excretion (mmol/24 h) was lower in the intervention period with borderline significance (157.3 ± 35.3 vs. 160.8 ± 32.5, *p* = 0.072), and the K excretion (mmol/24 h) was significantly higher in the intervention period (40.0 ± 9.7 vs. 38.0 ± 8.5, *p* = 0.001). In total, there was no significant difference in mean BMI (*p* = 0.410), but for women, it was significantly higher in the intervention period than in the control period (22.54 ± 3.65 vs. 22.46 ± 3.66, *p* = 0.039). The mean changes in BP and urinary Na/K were similar for those who had intervention meals in the first half and second half of the experimental period; the mean change (95% CI) in SBP (mmHg) was 0.51 (−1.01, 2.03) and 0.89 (−0.25, 2.03), *p* = 0.695, and the mean change in urinary Na/K (mmol/mmol) was −0.57 (−1.16, 0.01) and −0.36 (−0.71, −0.01), *p* = 0.531, for the first half and second half, respectively. The results were similar after adjusting for changes in BMI.

### 3.6. Taste Evaluation

The taste evaluation results for the control and intervention meals are shown in Table 5. There was no significant difference in taste evaluation for the intervention meals between men and women. For the control and intervention meals, 73.7% and 69.5% of the participants answered that the taste was fine, respectively, and the difference was not significant.

## 4. Discussion

We conducted a crossover intervention study at two cafeterias of a company in which lunches with low-Na/K seasonings and dairy were provided for 4 weeks, as well as ordinary lunches, showing that the mean urinary Na/K ratio significantly decreased from 4.10 to 3.69, with no significant difference in the BP level. In this study, we intended to evaluate the effects when relatively young, healthy participants in a free-living environment utilized a cafeteria offering intervention meals. They were not instructed to change their eating habits, such as reducing salt intake or having more vegetables. Several reasons may explain the lack of observed BP reduction. In previous intervention studies in which the participants were older hypertensives, it was reported that the use of low-Na/K salt had lowered BP levels [19,20,21]. The participants in this study were relatively young (mean age was 44.3 years), and more than half of the participants were normotensive (SBP < 120 mmHg and DBP < 80 mmHg). The BP-lowering effects of Na/K reduction were reported to be greater in elderly people and hypertensive individuals [34,35,36]. Herein, the intervention meals were provided only once a day on weekdays, and complete adherence to the intervention (five meals per week) was not achieved by all participants, which may have resulted in insufficient intervention intensity. Furthermore, the intervention effects may have been diluted on weekends when participants did not eat the intervention meals. The intervention period was also relatively short and may have been insufficient for the effects on BP to be observed. In the DASH Study, participants ate intervention meals for the whole day, 7 days a week, for 30 consecutive days [12]. Increasing the use of low-Na/K seasonings not only in workplace lunches but also in home cooking, dining out, and processed foods would enable further reduction of the dietary Na/K and potentially lower the BP by a significant degree.

We initially planned for 400 participants, but the number of individuals who agreed to participate was limited to 202 when office attendance was restricted during the COVID-19 pandemic, and only 166 participants cooperated with all survey procedures. This study may have been underpowered to detect the expected 1-mmHg SBP change. With a certain number of participants dropping out, an even greater number of enrollments was required. Moreover, the decrease in urinary Na/K ratio was smaller than expected. Therefore, the non-significant difference in the change in BP should be interpreted with caution. Another potential limitation of our study is the absence of a washout period between the intervention and control periods. In crossover trials with a washout, it is recommended to calculate the change from baseline within each period and compare these changes between the intervention and control conditions. In our design, however, the baseline of the second period may have been influenced by the preceding period, and we performed a direct comparison between the intervention and control periods in the present study. Consequently, the possibility of physiological or behavioral carryover effects cannot be ruled out. Future studies with a washout period will allow for baseline-adjusted analyses. Furthermore, we used paired t-tests and did not perform mixed-effects models that could account for repeated measurements, within-subject variability, and other factors. Further research with a sufficient number of participants is necessary to consider these factors.

In the INTERSALT cross-sectional study, where the BP and 24 h urinary Na and K excretions were measured from participants aged 20–59 years in 52 populations among 37 countries, the elevation of mean BP in older age groups in a population was suppressed in populations with low urinary Na/K ratios [9]. In this study, the urinary Na/K ratio decreased, but no reduction in BP was observed. However, maintaining a lower dietary Na/K ratio from an early age in normotensive individuals may prevent the subsequent development of hypertension.

The mean change in the urinary Na/K ratio (mmol/mmol) was −0.40 (−0.21 for men and −0.71 for women). Prior to conducting the intervention, we estimated that the mean dietary Na/K ratio would decrease by 0.8, which corresponded to a mean decrease of 1.0 in the urinary Na/K ratio [27], but the actual change was smaller. We expected that the participants would use the cafeteria 5 times/week, but the average number was 4.2 times/week, and the participants chose ordinary meals instead of low-Na/K meals in approximately 10% of their cafeteria visits. Overall, the average frequency of low-Na/K meals was 3.7 times/week (26% less than expected). This is because we did not require participants to strictly adhere to the low-Na/K meal every day in the cafeteria during the intervention period, allowing them free choice when leaving the workplace, traveling on business, or working from home, or because participants chose ordinary meals instead. While full compliance was not achieved, the results may have been closer to those in the actual working environment, and we believe this provided useful information for improving the dietary environment of working individuals.

Moreover, it is possible that the participants did not eat their entire meal, especially if they left some of the soup from the noodles. We did not conduct a leftover food survey due to time constraints among the employees who use the cafeteria, and thus, the actual amount of food left over during this study was unknown. There may have been a discrepancy between the Na and K content of cafeteria meals and the actual Na and K intake of participants. However, there were no significant differences in taste evaluations (fine, strong, light) between the low-Na/K meal and the control meal. Therefore, it was thought that the amount of soup a participant would leave or consume did not differ significantly between low-Na/K meals and control meals. Some participants may have consumed high-Na foods in other meals during the low-Na/K meal period or may have used more discretionary seasonings to compensate for the reduced Na intake. This might be based on Na appetite, which has been reported to physiologically regulate Na intake within a narrow range [37]. According to the estimated 24 h urinary Na and K excretions, Na excretion decreased by 2.2% and K excretion increased by 5.3% in the intervention period, compared with the control period, resulting in a 10% decrease in urinary Na/K. K excretion increased more than the decrease in Na excretion, which may indicate a greater contribution of dairy consumption than the use of the low-Na/K seasonings to the decrease in urinary Na/K.

The same amount of food was served to men and women in the cafeterias, and the decrease in urinary Na/K ratio was more apparent in women (−0.71) than in men (−0.21). The overall dietary intake was higher in men than in women, and the contribution of lunch in the cafeteria may have been higher for women than for men. Moreover, it has been reported that women often choose healthier diets, including low-salt meals, than men [38]. A previous study among company employees showed that 54.9% of women and 38.1% of men answered that they were interested in salt reduction [39]. There is a possibility that women complied more closely with the intervention. These factors may have interacted and influenced the results. Meanwhile, most of both men and women reported that the taste of low-Na/K meals was fine (73.5% of men and 62.5% of women). The results may indicate the potential for a decrease in dietary Na/K among men by expanding the use of low-Na/K seasonings in various settings outside the employee cafeteria.

The overall decrease in mean urinary Na/K ratio was modest (−0.40), and SBP did not show a statistically significant change. However, even a small decrease in the mean urinary Na/K ratio could be associated with a decrease in BP. Urinary Na/K ratio was measured annually over a two-year period to examinees in regular health checkups in a city in Japan [10]. Urinary Na/K ratio was provided to the participants at the health checkup venue along with teaching materials. The average urinary Na/K ratio was significantly lower in the second year than in the first year (5.4 ± 3.0 to 4.9 ± 2.2, *p* < 0.01), and the mean SBP in the second year (130.9 ± 17.4 mmHg) was lower than that in the first year (132.1 ± 17.9 mmHg) (*p* = 0.04). The educational effect on the examinees may be associated with a decrease in urinary Na/K ratio and BP one year later. In our current study, the urinary Na/K ratio improved to a similar degree without educating participants; they simply consumed cafeteria lunch and continued their usual eating habits outside the company cafeteria. Even a one-meal-per-day intervention, if sustained over a long period, could potentially be associated with a decrease in BP and, eventually, reduction in long-term cardiovascular risk at the population level.

The estimated changes in estimated 24 h urinary excretions in Na (~3 mmol reduction) and K (~2 mmol increase) were modest. The small but directionally favorable changes in urinary Na and K suggest partial adherence and demonstrate that modest dietary modifications are achievable without compromising acceptability. Future studies with a longer duration or wider use of low-Na/K seasonings may be required to elicit measurable BP changes.

Approximately 100 kcal of energy was supplied from a pack of milk/yogurt during the intervention, and a slight but significant increase in BMI of 0.08 kg/m^2^ (0.2 kg in body weight) was observed in women after the intervention. Dairy products are efficient foods for lowering the dietary Na/K ratio, but when habitually increasing it, other food intake with corresponding energy may need to be reduced to maintain body weight. For some participants, the consumption of dairy at the cafeteria may have discouraged the consumption at home in compensation. According to the SDPQ collected at baseline and the last visit, the increase in dairy intake was not as large as expected (Supplementary Table S2). However, this questionnaire asked about food consumption “in the last 1–2 months”, and it was not sufficiently detailed to adequately reflect changes over the study periods (4 weeks of intervention meal and 4 weeks of control meal). It was necessary to conduct a more sensitive survey, such as asking about dairy consumption weekly, separating consumption in and out of the employee cafeteria. Unlike interventions that use active drugs and placebos, it may be necessary to consider whether the intake of the intervention food causes a decrease in the other regular meals, particularly in dietary interventions that use ordinary foods. Because there is a possibility that when provided with more food than they normally have, some people might think they have had enough and skip foods they would normally eat spontaneously.

We had calculated the average NaCl content of the cafeteria lunch to be 5 g/serving based on the ingredient list before the study [28], but it was 4.3 g (74.2 mmol) for the Japanese set menu, and 4.7 g (80.4 mmol) for noodles, according to the measured values from the sampled foods. We did not ask the cafeteria manager to use predetermined amounts of seasoning; instead, the amount was decided based on their subjective taste of the dishes. Hence, we could not accurately assess the exact amounts of seasonings used in either the intervention meals or the ordinary meals. The actual mean reduction in NaCl in the intervention meals was 0.4–0.5 g (7–9 mmol of Na) per serving, which corresponded to approximately 10% of the control meals. Notably, the NaCl reduction was smaller than the predicted value of 0.8 g [28]. This may have been attributed to the relatively high use of low-Na/K seasonings that contain 25% less NaCl compared with ordinary seasonings. In our previous study in which participants used the low-Na/K seasonings in home cooking, participants used each seasoning approximately 10% to 20% more than the ordinary seasonings [31]. Using higher amounts of low-Na/K seasonings than the ordinary seasonings may have also contributed to the favorable taste evaluation for low-Na/K meals, thereby avoiding a thin flavor. Further research is needed to establish standardized usage levels for low-Na/K seasonings that deliver equivalent flavor to the ordinary seasonings, which will enable more relevant estimates of effects such as urinary Na/K ratio. Around 70% of participants reported a good taste for both the low-Na/K and ordinary meals in the final survey, without a significant difference. We were unable to standardize the amount of seasoning used, but we could standardize the taste and verified the effects of using low-Na/K seasonings. We think these results will also be useful for actual restaurants that prioritize sales.

Milk and yogurt are thought to be useful for lowering the dietary Na/K ratio because they contain approximately 300 mg of K per serving and do not require Na-containing seasonings for consumption. They are also high in calcium and magnesium, which have antihypertensive effects [40,41,42]. The average intake of milk in Japanese adults in 2019 was shown to be relatively low, 110.7 g/day [6], which is one reason for the low K intake in Japanese people [43]. Although the high frequency of lactose intolerance in Asia [44] may contribute to their low milk intake, no person refused to participate or discontinued participation because of abdominal symptoms with milk consumption in this study. It was considered useful to recommend approximately one cup of milk/yogurt daily in the Japanese adult population, as a food that lowers dietary Na/K and contributes to the prevention of hypertension. People can obtain ~300 mg of K from one cup of milk/yogurt, which involves less effort as well as cost than having fruits and vegetables.

Using low-Na/K seasonings allowed the dietary Na/K ratio to decrease without changing the user’s taste evaluation and without requiring additional work in the kitchen. Although the importance of Na reduction is widely understood, actual Na reduction is difficult to achieve, especially in populations where average Na intake has already decreased to a certain extent [45]. In the food industry, there is a widespread concern that reducing salt content will lead to a decline in sales [46,47]. When cooking home-cooked meals with less salt, families may not accept the less salty foods [48,49]. In the present study, it was shown that low-Na/K seasonings can be used without changing the taste evaluation while achieving a significant decrease in urinary Na/K.

The WHO reported a NaCl intake target of 5.0 g/day and a K intake target of 3500 mg/day [7,8], but consumption is far from the targets in many countries around the world [50]. For the past decade, the average intake of NaCl and K for Japanese adults has been around 10 g/day and 2200 mg/day, respectively, and there has been almost no shift toward improvement [6]. Meanwhile, the use of processed foods and restaurant-based diets is growing in many countries, making it more difficult to reduce Na, which is an inexpensive seasoning, and increase K, for which the major food sources are vegetables and fruits. Low-Na/K seasonings may offer a promising way out of this situation to achieve a hypotensive food environment.

This study has some more limitations. This study is limited in its generalizability because the results were obtained from the employees of one company located in a metropolitan area, who used cafeterias operated by a food service company. They were also relatively young, and the proportion of employees with hypertension was low. The decrease in urinary Na/K was smaller than expected, and there were no significant differences in BP between control and intervention meal periods. The effects of moderate dietary Na/K reduction on BP in early middle-aged individuals should be further investigated in future studies. Both men and women were provided with the same amount of cafeteria meals, and changes in urinary Na/K ratio differed between genders. Therefore, generalizing these results to populations with different male-to-female ratios has limitations. The cafeteria menu consisted mainly of Japanese set menus and Japanese-style noodles in which a high amount of high-Na seasonings was used. The high use of high-Na seasonings is one of the characteristics of the Japanese diet, whereas Western menus generally use less [27,51]. The effect of using low-Na/K seasonings may be different in cafeterias with mainly Western-style menus.

## 5. Conclusions

Providing lunch using low-Na/K seasonings and dairy in company cafeterias used by relatively young, normotensive persons significantly decreased the mean Na/K ratio in the spot urine of the participants, with no change in BP. Participants were not instructed to change their diet outside the cafeteria. There was no burden associated with the intervention on either the kitchen or the users, and the reduction in dietary Na/K ratio with low-Na/K seasonings and dairy was thought to be sustainable without burden. Widespread use of low-Na/K seasonings and habitual consumption of dairy might be a practical approach to sustainably decrease dietary Na/K and mitigate age-related increases in BP.

## Figures and Tables

**Table 1:** Characteristics of the participants.

		Men (*n* = 102)		Women (*n* = 64)		*p*	Total (*n* = 166)	
		*n*	(%)	*n*	(%)		*n*	(%)
Age (years), mean (SD)		44.8	(11.3)	43.5	(12.0)	0.493	44.3	(11.5)
Age group	22–39 years	35	(34.3)	26	(40.6)	0.210	61	(36.7)
	40–49 years	28	(27.5)	10	(15.6)		38	(22.9)
	50–66 years	39	(38.2)	28	(43.8)		67	(40.4)
Awareness of hypertension ^a^		29	(28.4)	9	(14.1)	0.032	38	(22.9)
Use of antihypertensives		13	(12.7)	3	(4.7)	0.087	16	(9.6)
Smoke	Current smoker	16	(15.7)	4	(6.3)	0.001	20	(12.0)
	Never smoker	57	(55.9)	57	(89.1)		114	(68.7)
	Ex-smoker	29	(28.4)	3	(4.7)		32	(19.3)
Having a low-salt diet		20	(19.6)	10	(15.6)	0.516	30	(18.1)
Milk/yogurt consumption ^b^, *n* (%)								
Do not consume		16	(15.7)	6	(9.4)	0.556	22	(13.3)
≤1 cup/week		18	(17.6)	11	(17.2)		29	(17.5)
2–3 cups/week		17	(16.7)	16	(25.0)		33	(19.9)
4–5 cups/week		15	(14.7)	7	(10.9)		22	(13.3)
1 cup/day		28	(27.5)	21	(32.8)		49	(29.5)
≥2 cups/day		8	(7.8)	3	(4.7)		11	(6.6)
Cafeteria use Cafeteria A		61	(59.8)	21	(32.8)	0.001	82	(49.4)
Cafeteria B		41	(40.2)	43	(67.2)		84	(50.6)
Cafeteria use (/week), mean (SD)		4.2	(1.2)	3.6	(1.3)	0.002	4	(1.3)

SD, standard deviation. ^a^ have ever been diagnosed with hypertension or using antihypertensives. ^b^ Obtained from the short dietary propensity questionnaire [29]. *p*-values were obtained from *t*-tests for continuous variables and chi-squared tests for categorical variables.

**Table 2:** Results of the baseline survey.

	Men		Women		*p*	Total	
	Mean	(SD)	Mean	(SD)		Mean	(SD)
SBP (mmHg)	120.6	(12.8)	108.0	(16.2)	<0.001	115.7	(15.4)
DBP (mmHg)	79.0	(11.1)	70.6	(11.6)	<0.001	75.8	(12.0)
BP category (including participants using antihypertensives) ^a^							
Normal BP (SBP < 120 and DBP < 80), *n* (%)	45	(44.1)	49	(76.6)	0.001	94	(56.6)
High normal BP (SBP 120–129 and/or DBP < 80), *n* (%)	11	(10.8)	1	(1.6)		12	(7.2)
Elevated BP (SBP 130–139 and/or DBP 80–89), *n* (%)	32	(31.4)	11	(17.2)		43	(25.9)
Hypertension (SBP ≥ 140 and/or DBP ≥ 90), *n* (%)	14	(13.7)	3	(4.7)		17	(10.2)
Hypertension ^b^, *n* (%)	23	(22.5)	6	(9.4)	0.001	29	(17.5)
BMI (kg/m^2^)	24.2	(3.1)	22.5	(3.6)	0.002	23.5	(3.4)
Estimated 24 h urinary Na excretion ^c^ (mmol/24 h)	160.1	(31.6)	144.5	(25.9)	0.001	154.1	(30.4)
Estimated 24 h urinary K excretion ^c^ (mmol/24 h)	38.7	(9.0)	33.8	(6.5)	<0.001	36.8	(8.4)
Urinary Na/K (mmol/mmol)	4.01	(1.89)	4.20	(2.00)	0.549	4.09	(1.93)

SD, standard deviation; SBP, systolic blood pressure; DBP, diastolic blood pressure; BMI, body mass index. ^a^ based on the classification reported in the Japanese Society of Hypertension Guidelines for the Management of Hypertension (JSH 2019 [33]). ^b^ have ever been diagnosed with hypertension or using antihypertensives. ^c^ Obtained from Tanaka’s formula [32].

**Table 3:** Na and K contents and Na/K ratio in dishes served for control and intervention meals, between 23 January and 17 March 2023 (40 business days).

	Na (mmol/Serving)			K (mmol/Serving)			Na/K Ratio (mmol/mmol)		
	Mean	(SD)	*p*	Mean	(SD)	*p*	Mean	(SD)	*p*
Main dish for the Japanese set menu									
Control	47.9	(23.6)	0.013	8.4	(3.4)	<0.001	6.3	(3.5)	<0.001
Intervention	40.0	(18.7)		11.5	(3.5)		3.5	(1.3)	
Miso soup for the Japanese set menu									
Control	26.8	(3.4)	0.413	1.4	(0.2)	<0.001	19.2	(2.3)	<0.001
Intervention	26.4	(4.0)		3.5	(0.6)		7.7	(1.2)	
Japanese set menu (main dish + miso soup)									
Control	74.2	(23.6)	0.008	9.9	(3.4)	<0.001	8.2	(3.1)	<0.001
Intervention	66.4	(18.4)		15.1	(4.7)		4.6	(1.2)	
Noodle (soup and topping)									
Control	80.4	(14.8)	0.008	4.1	(1.5)	<0.001	22.7	(11.3)	<0.001
Intervention	71.8	(14.7)		11.8	(3.4)		6.6	(2.6)	
Dairy (provided in the intervention meal period) ^a^									
Regular milk (200 mL)	3.8			7.9			0.5		
Low-fat milk (200 mL)	4.1			9.2			0.4		
Low-fat yogurt (200 mL)	3.9			8.5			0.5		

^a^ data provided by the manufacturer.

**Table 4:** Cafeteria use, spot urine measurement, and blood pressure in the control and intervention periods (166 participants, 102 men and 64 women).

	Control		Intervention		Difference (Intervention—Control)			*p*
	Mean	(SD)	Mean	(SD)	Mean	(SE)	(95%CI)	
Cafeteria use (/week)								
Men	4.4	(0.9)	4.3	(1.0)	−0.07	(0.1)	(−0.18, 0.04)	0.232
Women	3.9	(1.2)	4.0	(1.1)	0.13	(0.1)	(−0.08, 0.35)	0.216
Total	4.2	(1.1)	4.2	(1.0)	0.01	(0.1)	(−0.10, 0.11)	0.868
Use of dairy at the cafeteria in the intervention meal period (/week)								
Men			4.3	(1.2)				
Women			4.1	(1.4)				
Total			4.2	(1.3)				
BMI (kg/m^2^)								
Men	24.1	(3.1)	24.1	(3.0)	0.0	(0.0)	(−0.1, 0.0)	0.471
Women	22.46	(3.66)	22.54	(3.65)	0.08	(0.03)	(0.00, 0.15)	0.039
Total	23.5	(3.4)	23.5	(3.3)	0.0	(0.0)	(−0.0, 0.1)	0.410
SBP (mmHg)								
Men	120.5	(10.8)	121.0	(11.8)	0.6	(0.6)	(−0.6, 1.8)	0.333
Women	108.2	(13.9)	109.1	(15.6)	0.9	(0.8)	(−0.7, 2.49)	0.261
Total	115.7	(13.4)	116.4	(14.6)	0.7	(0.5)	(−0.2, 1.5)	0.141
DBP (mmHg)								
Men	78.8	(9.6)	79.2	(9.6)	0.4	(0.5)	(−0.5, 1.3)	0.371
Women	72.0	(9.4)	72.9	(10.7)	0.9	(0.5)	(−0.2, 2.0)	0.100
Total	76.2	(10.0)	76.8	(10.5)	0.6	(0.4)	(−0.1, 1.3)	0.089
Urinary Na/K ratio (mmol/mmol)								
Men	4.15	(2.20)	3.94	(2.13)	−0.21	(0.19)	(−0.59, 0.16)	0.266
Women	4.02	(2.24)	3.31	(1.39)	−0.71	(0.24)	(−1.20, −0.22)	0.005
Total	4.10	(1.89)	3.69	(1.76)	−0.40	(0.15)	(−0.11, −0.70)	0.008
Estimated 24 h urinary Na excretion (mmol/24 h)								
Men	168.2	(32.8)	167.2	(34.1)	−1.0	(2.5)	(−6.1, 4.0)	0.686
Women	149.1	(28.7)	141.7	(31.5)	−7.4	(3.0)	(−1.5, −13.3)	0.015
Total	160.8	(32.5)	157.3	(35.3)	−3.5	(1.9)	(−7.3, 0.3)	0.072
Estimated 24 h urinary K excretion (mmol/24 h)								
Men	39.6	(9.1)	41.9	(10.5)	2.2	(0.2)	(0.7, 3.7)	0.005
Women	35.3	(6.5)	36.9	(7.2)	1.6	(0.8)	(0.0, 3.2)	0.047
Total	38.0	(8.5)	40.0	(9.7)	2.0	(0.6)	(0.9, 3.1)	0.001

SD, standard deviation; SE, standard error; CI, confidence interval; BMI, body mass index; SBP, systolic blood pressure; DBP, diastolic blood pressure. *p*-values were obtained from paired *t*-tests.

**Table 5:** Taste evaluation of the control and intervention meals, reported at the last visit.

	Men		Women		*p*^a^ (Men vs. Women)	Total	
	*n*	(%)	*n*	(%)		*n*	(%)
Control meal							
Fine	79	(82.3)	33	(58.9)	0.001	112	(73.7)
Strong	13	(13.5)	22	(39.3)		35	(23.0)
Light	4	(4.2)	1	(1.8)		5	(3.3)
Intervention meal							
Fine	72	(73.5)	35	(62.5)	0.117	107	(69.5)
Strong	21	(21.4)	20	(35.7)		41	(26.6)
Light	5	(5.1)	1	(1.8)		6	(3.9)
*p*^b^ (control vs. intervention)	0.122		0.819			0.277	

^a^ Obtained from the chi-squared test. ^b^ Obtained from the McNemar–Bower test.

## Data Availability

The data are not available in open-access form because participants did not consent to their data being made available in this way.

## Supplementary Materials

The following supporting information can be downloaded at: https://www.mdpi.com/article/10.3390/nu17243856/s1, Table S1: Choice of meal and dairy per week according to food choice sheets given in the cafeteria in intervention period; Table S2: Frequency of milk/yogurt consumption in the last 1–2 months according to a Short Dietary Propensity Questionnaire from the surveys at baseline and the last visit, for cafeteria A users (intervened in the first 4 weeks) and cafeteria B users (intervened in the second 4 weeks).

## Abbreviations

The following abbreviations are used in this manuscript:

BP	Blood pressure
CVD	Cardiovascular disease
WHO	World Health Organization
SDPQ	Short dietary propensity questionnaire
SBP	Systolic blood pressure
DBP	Diastolic blood pressure
BMI	Body mass index
