# Supplementary material for: Intervention Using Low-Na/K Seasonings and Dairy at Japanese Company Cafeterias as a Practical Approach to Decrease Dietary Na/K and Prevent Hypertension
Source: Nutrients. 2025 Dec 10;17(24):3856. doi: 10.3390/nu17243856 (PMC12735755; doi:10.3390/nu17243856)
Supplement: Supplementary file 1 [file nutrients-17-03856-s001.zip › nutrients-4001167-supplementary.pdf]

Table S1. Choice of meal and dairy per week according to food choice sheets given in the cafeteria in intervention period.

	Men		Women		*P*	Total	
	Mean	(SD)	Mean	(SD)		Mean	(SD)
Total use of cafeteria meal (/week)	3.7	(1.2)	3.7	(1.0)	0.979	3.7	(1.2)
Total of low-Na/K meal	3.3	(1.3)	3.3	(1.1)	0.717	3.3	(1.2)
Japanese-style set menu	1.9	(1.3)	1.9	(1.0)	0.740	1.9	(1.2)
Noodle	1.5	(1.2)	1.3	(1.1)	0.489	1.4	(1.2)
Other menu with ordinary seasoning	0.3	(0.5)	0.4	(0.5)	0.452	0.4	(0.5)
Use of dairy at cafeteria (/week)							
Total dairy	3.6	(1.3)	3.5	(1.1)	0.658	3.6	(1.2)
Regular milk	0.4	(1.0)	0.3	(0.8)	0.247	0.4	(0.9)
Low-fat milk	0.1	(0.5)	0.1	(0.3)	0.774	0.1	(0.6)
Low-fat yoghurt	3.1	(1.6)	3.2	(1.3)	0.699	3.1	(1.5)

SD, standard deviation

P values obtained from t-tests.

Table S2. Frequency of milk/yoghurt consumption in the last 1-2 months according to a Short Dietary Propensity Questionnaire from the surveys at baseline and the last visit, for cafeteria A users (intervened in the first 4 weeks) and cafeteria B users (intervened in the second 4 weeks).

	Baseline		Last visit		*P*
	n	(%)	n	(%)	
Total					
Do not consume	22	(13.3)	2	(1.2)	0.005
≤1 cup/wk	29	(17.6)	31	(18.7)	
2-3 cups/wk	33	(20.0)	37	(22.3)	
4-5 cups/wk	21	(12.7)	20	(12.0)	
1 cup/day	49	(29.7)	60	(36.1)	
≥ 2 cups/day	11	(6.7)	16	(9.6)	
Cafeteria A user (intervened in the first 4 wks)					
Do not consume	14	(17.3)	1	(1.2)	0.012
≤1 cup/wk	18	(22.2)	23	(28.0)	
2-3 cups/wk	17	(21.0)	21	(25.6)	
4-5 cups/wk	13	(16.0)	8	(9.8)	
1 cup/day	15	(18.5)	25	(30.5)	
≥ 2 cups/day	4	(4.9)	4	(4.9)	
Cafeteria B user (intervened in the second 4th wks)					
Do not consume	8	(9.5)	1	(1.2)	0.254
≤1 cup/wk	11	(13.1)	8	(9.5)	
2-3 cups/wk	16	(19.0)	16	(19.0)	
4-5 cups/wk	8	(9.5)	12	(14.3)	
1 cup/day	34	(40.5)	35	(41.7)	
≥ 2 cups/day	7	(8.3)	12	(14.3)	

P values obtained from McNemar-Bowker tests.
